# Hydrothermal Synthesis and Upconversion Properties of About 19 nm Sc_2_O_3_: Er^3+^, Yb^3+^ Nanoparticles with Detailed Investigation of the Energy Transfer Mechanism

**DOI:** 10.1186/s11671-018-2794-9

**Published:** 2018-11-22

**Authors:** Fen Li, Jing Li, Li Chen, Yuxin Huang, Yaru Peng, Yongshi Luo, Ligong Zhang, Jiajia Mu

**Affiliations:** 1 School of Chemical Engineering & Advanced Institute of Materials Science, Changchun University of Technology, 2055 Yan’an Street, Changchun, 130012 Jilin China; 2 School of Materials Science and Engineering, Changchun University of Technology, 2055 Yan’an Street, Changchun, 130012 Jilin China; 3 State Key Laboratory of Luminescence and Applications, Changchun Institute of Optics, Fine Mechanics and Physics, Chinese Academy of Sciences, 3888 Eastern South Lake Road, Changchun, 130033 China; 4 School of Science, Beihua University, 15 Jilin Street, Jilin, 132013 Jilin China

**Keywords:** Sc_2_O_3_, Hydrothermal synthesis, Upconversion, Energy transfer, Er^3+^/Yb^3+^

## Abstract

The Sc_2_O_3_: Er^3+^, Yb^3+^ nanoparticles (NPs) with the size of about 19 nm were synthesized by a simple oleic acid-mediated hydrothermal (HT) process. X-ray diffraction (XRD), transmission electron microscopy (TEM), upconversion luminescence (UCL) spectra, and decay curves were used to characterize the resulting samples. The Sc_2_O_3_: Er^3+^, Yb^3+^ NPs made by HT method exhibit the stronger UCL, of which the red UCL are enhanced by a factor of 4, in comparison with those samples prepared by solvothermal (ST) method at the same optimized lanthanide ion concentrations. The UCL enhancement can be attributed to the reduced surface groups and longer lifetimes. Under 980 nm wavelength excitation, the decay curves of Er^3+^: (^2^H_11/2_, ^4^S_3/2_) → ^4^I_15/2_ and ^4^F_9/2_ → ^4^I_15/2_ emissions for Sc_2_O_3_: Er^3+^, Yb^3+^ NPs samples are both close to each other, resulting from the cross relaxation energy transfer from Er^3+^ to Yb^3+^, followed by an energy back transfer within the same Er^3+^-Yb^3+^ pair. Also, under the relatively low-power density, the slopes of the linear plots of log(*I*) vs. log(*P*) for red and green emissions are 2.5 and 2.1, implying the existence of three-photon processes. Our results indicate that Sc_2_O_3_: Er^3+^, Yb^3+^ NPs is an excellent material for achieving intense UCL with small size in the biological fields.

## Introduction

Infrared to visible upconversion luminescence (UCL) has been extensively studied for its fundamental value [1–3] and its various potential applications in upconversion lasers, bioimaging, infrared imaging, solar cells, etc. [4–8]. The co-doping of Er^3+^ and a high concentration of sensitizer Yb^3+^ forms the most attractive energy transfer (ET) upconversion system [1]. Under 980 nm infrared excitation of the sensitizer Yb^3+^, this system can generate green and red emission originating from the (^2^H_11/2_, ^4^S_3/2_) → ^4^I_15/2_ and ^4^F_9/2_ → ^4^I_15/2_ transitions of Er^3+^, respectively [9]. Selection of appropriate host material is essential in the synthesis of lanthanide-doped nanocrystals (NCs) with favorable optical properties such as high UC efficiency and controllable emission profile. The practical applications require the development of more efficient, high stability UC materials with low excitation density [10, 11]. Oxide materials are usually very stable chemically, mechanically, and thermally, and could therefore be promising hosts for UC applications [3, 12–16]. The cubic sesquioxide materials (such as Y_2_O_3_, Lu_2_O_3_, Sc_2_O_3_, etc.) display particular structural characteristics and physical properties. For example, Y_2_O_3_ shows up the outstanding UCL as the typical oxide host [3, 17]. The Sc_2_O_3_ has the smallest lattice parameter. The short Sc–Sc bond length in Sc_2_O_3_ can produce the short distance within an Yb^3+^-Er^3+^ pair, speeding up the Yb^3+^ → Er^3+^ energy transfer. In our previous work, Sc_2_O_3_: Er^3+^, Yb^3+^ nanostructures were obtained using a biphasic solvothermal (ST) method [17]. The red UCL in this samples are enhanced, compared with the bulk sample synthesized using a solid-state (SS) reaction. The average crystal size of nanostructures has reduced to about 200 nm, which favors the application in fluorescence imaging.

A variety of chemical techniques, including coprecipitation, solvothermal synthesis (ST), hydrothermal method (HT), sol–gel processing, thermal decomposition, etc., have been demonstrated to synthesize lanthanide-doped UC NCs [14, 18–22]. Optimization of synthesis procedure is critical to obtain NCs with tailored crystal size, morphology, surface functionalization, and optical properties. The HT approach is a good choice due to its convenience, exemption from pollution, and the possibility of achieving satisfying crystallinity at a relatively low temperature [23]. Zhao et al. utilized an oleic acid-mediated HT method for the synthesis of UC NaYF_4_ nanorods, nanotubes, and flower-patterned nano-disks [20]. Chen et al. prepared Fe^3+^ co-doped NaYF_4_: Er, Yb UC NCs by a HT method using oleic acid as a capping ligand and a surface modifier [24]. In this work, Sc_2_O_3_: Er^3+^, Yb^3+^ nanoparticles (NPs) of 19 nm in average diameters have been first synthesized through a simple oleic acid-mediated HT method. We found the stronger UCL in this Sc_2_O_3_: Er^3+^, Yb^3+^NPs samples, of which the red UCL are enhanced by a factor of 4, in comparison with that in the same optimized concentration Sc_2_O_3_ samples by ST method. The UCL enhancement can be attributed to the reduced surface groups and longer lifetimes. Additionally, the UCL property and mechanism of HT-Sc_2_O_3_: Er^3+^, Yb^3+^ NPs were investigated by the spectra distribution, power dependence, and lifetime measurement.

## Experimental

### Sample Preparation

The Sc_2_O_3_: Er^3+^, Yb^3+^ samples were prepared by the HT method via the hydrolysis of relevant mineral salts in an ethanol scheme. The high purity raw materials of Sc_2_O_3_, Er_2_O_3_, and Yb_2_O_3_ powers were dissolved in dilute HNO_3_ and deionized water to obtain cationic nitrates solutions, respectively. The Sc(NO_3_)_3_, Er(NO_3_)_3_, and Yb(NO_3_)_3_ solutions with corresponding mole ratios were dissolved in absolute ethanol (20 ml), stirring to form a homogeneous solution. Then an aqueous sodium hydroxide solution (2 ml) was added dropwise to the above mixture with stirring for 30 min, followed by adding oleic acid (1 ml), then vigorous stirring for 1 to 2 h. The resulting suspension was placed in a close Teflon-lined stainless steel autoclave with 50 ml capacity and heated at 180 °C for 24 h. After the autoclave was cooled to room temperature, naturally the precipitate was then centrifuged and washed several times with deionized water and absolute ethanol, respectively. The powder was obtained after being dried in a vacuum oven at 80 °C for 15 h and annealed 700 °C for 2 h. For comparison, we prepared Sc_2_O_3_ samples prepared by the ST method at the same sintering temperature 700 °C for 2 h [17].

### Measurements and Characterization

Powder X-ray diffraction (XRD) datum was collected using Cu-Kα radiation (λ = 1.54056 Å) on an X-ray powder diffractometer (Rigaku D/Max IIA). Transmission electron microscopy (TEM) image was obtained by using a transmission electron microscope (JEM-2000EX) operating at an acceleration voltage of 200 kV. The UCL spectra were recorded with a spectrophotometer (Hitachi F-7000) and infrared spectra were performed by using a Triax 550 spectrometer (Jobin-Yvon) pumped with a power-controllable 980 nm diode laser at room temperature. Infrared spectra in transmission mode were measured on a Thermofisher Nicolet IS50 FT-IR spectrometer, using pressed KBr tablets. In fluorescence lifetime measurements, an optical parametric oscillator (OPO) was tuned to 980 nm as an excitation source, and the signals were detected by a Tektronix digital oscilloscope (TDS 3052).

## Results and Discussion

**Fig. 1:**
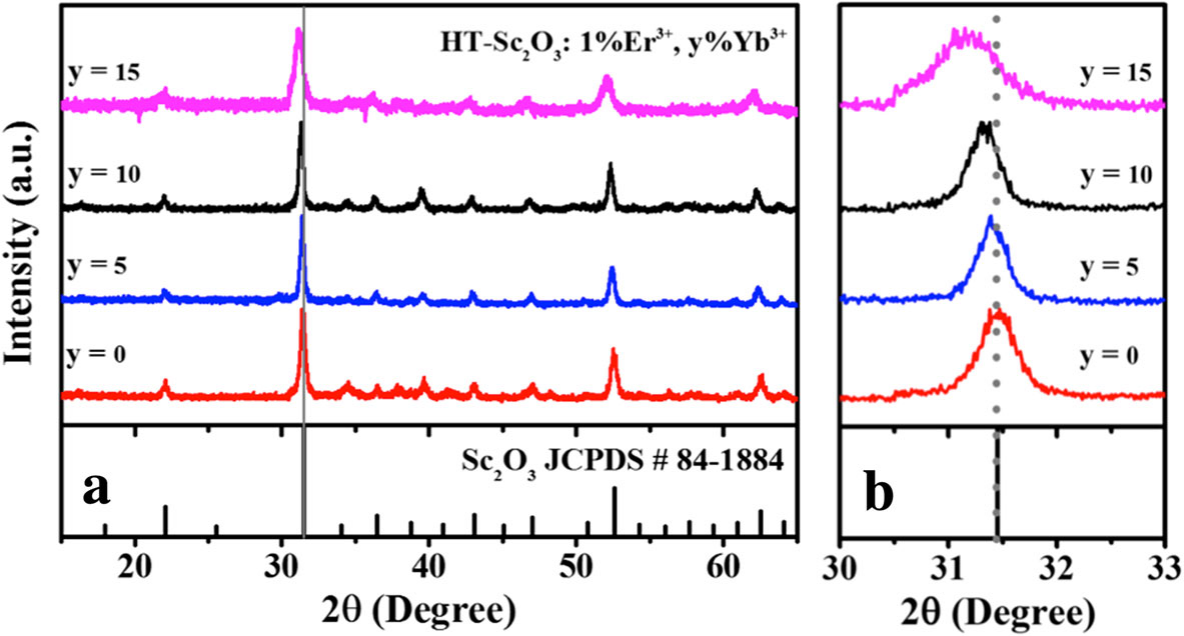
**a** XRD patterns for HT-Sc_2_O_3_: 1%Er^3+^, y%Yb^3+^ (*x* = 0, 5, 10, 15) NPs. **b** Magnified patterns in the diffraction angle ranged from 30° to 33°

**Fig. 2:**
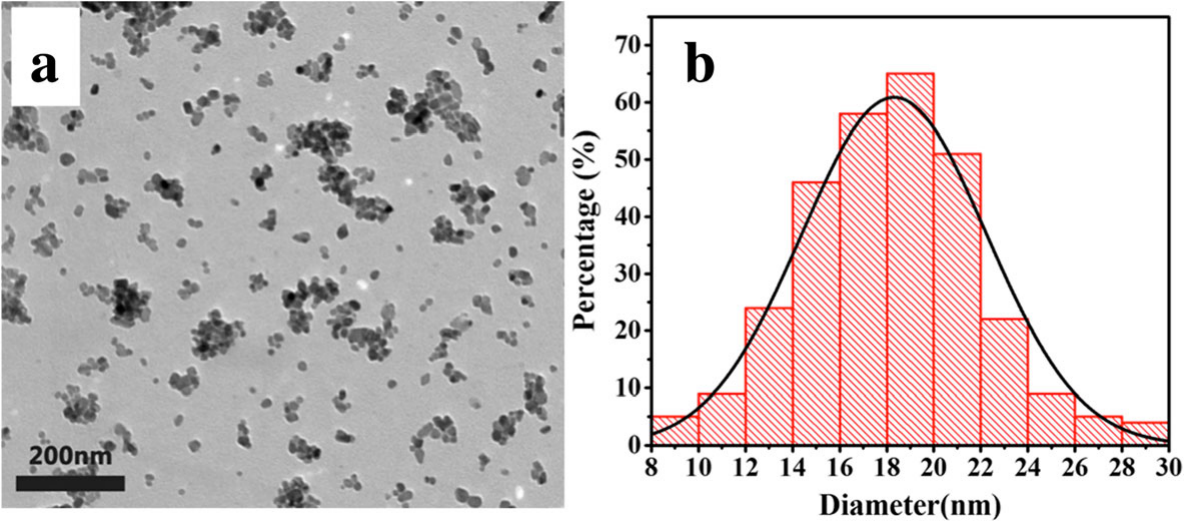
**a** TEM image and **b** histogram of size distribution of HT-Sc_2_O_3_: 1%Er^3+^, 5%Yb^3+^ NPs

The structures characterized by the XRD patterns are shown in Fig. 1a for samples by HT method with the nominal compositions of Sc_2_O_3_: 1%Er^3+^, y%Yb^3+^ (*x* = 0, 5, 10, 15). The pure phase Sc_2_O_3_ was synthesized in agreement with JCPDS card 84-1884. The host lattice exhibits the mineral bixbyite structure with the $Ia\overline{3}$ (T^h^_2_) symmetry [25]. In this structure, Sc^3+^ is sixfold with the effective ionic radius (0.745 Å). The Yb^3+^ ions owned the large ionic radius (0.868 Å) occupy Sc^3+^ sites to expand the lattice cell volume, making XRD peaks shift to smaller angles as Yb^3+^ concentration increases as shown in the magnified patterns of Fig. 1b. To further reveal the morphology and size distribution, the as-prepared Sc_2_O_3_ samples were characterized by TEM. Figure 2a shows the TEM image of HT-Sc_2_O_3_: 1%Er^3+^, 5%Yb^3+^. We obtained the sphered NPs with relatively uniform size and good monodispersity. Figure 2b depicts the histogram of the size distribution; these data were obtained from the TEM image of more than 300 NPs. The average diameter of NPs was determined to be about 19 nm.

**Fig. 3:**
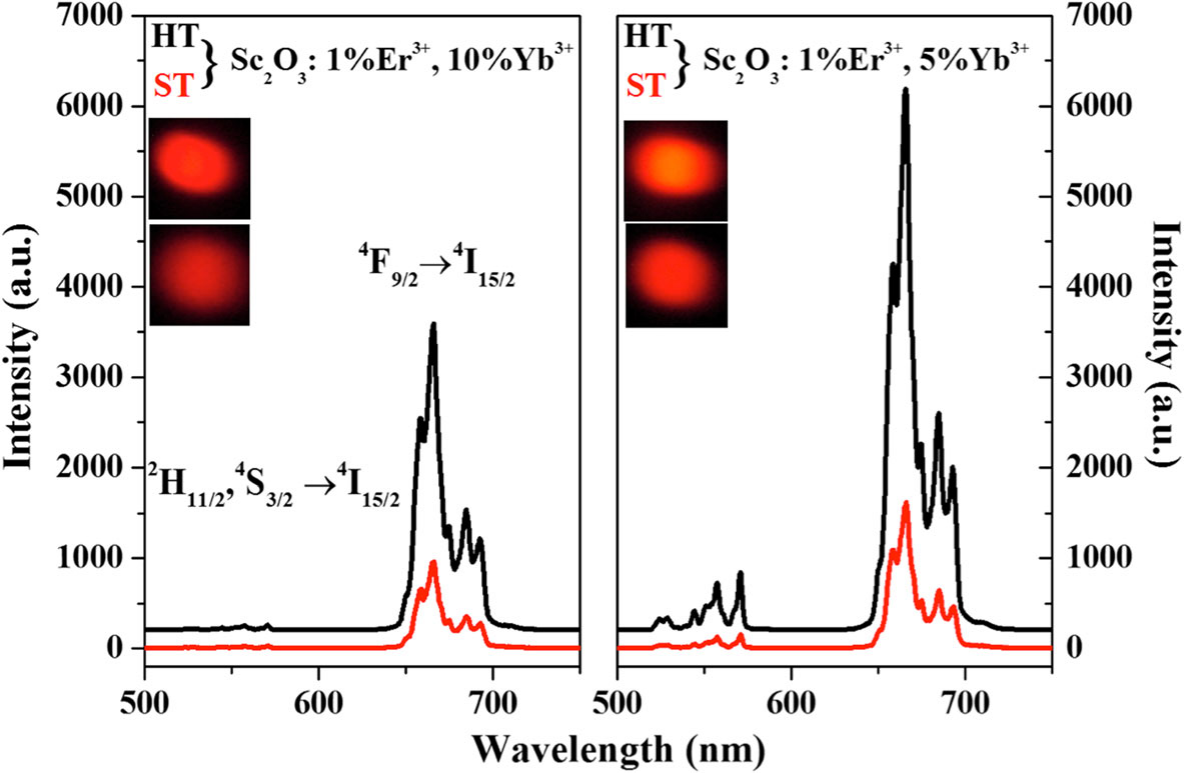
UCL spectra of Sc_2_O_3_: 1%Er^3+^, 10%Yb^3+^ (**a**) and Sc_2_O_3_: 1%Er^3+^, 5%Yb^3+^ (**b**) samples prepared by HT and ST methods, respectively, pumped under 980 nm excitation. The insets present the digital photographs of corresponding samples

Figure 3 shows the UCL spectra of Sc_2_O_3_: 1%Er^3+^, 10%Yb^3+^ (a) and Sc_2_O_3_: 1%Er^3+^, 5%Yb^3+^ (b) samples prepared by HT and ST methods under 980 nm excitation with an output power density of 3 mW mm^−2^. The strong emission bands centered at ~ 550 and 660 nm are attributed to the 4*f* - 4*f* electronic transitions of Er^3+^: (^2^H_11/2_, ^4^S_3/2_) → ^4^I_15/2_ and ^4^F_9/2_ → ^4^I_15/2_ transitions, respectively. The insets present the digital photographs of corresponding samples. It reveals that UCL has been dramatically enhanced for the HT sample, compared with the ST one. For HT-Sc_2_O_3_ samples, the calculated enhancement factor of red UCL is around 4, compared with corresponding ST-Sc_2_O_3_ samples. It is known that the size of samples has an influence on UCL intensity, which decreased with the decreasing of the size. However, for HT-Sc_2_O_3_ sample, it owns smaller size and more intensive UCL. It indicates the HT-Sc_2_O_3_ sample is an excellent material owned intense UCL with small size for the biological fields.

**Fig. 4:**
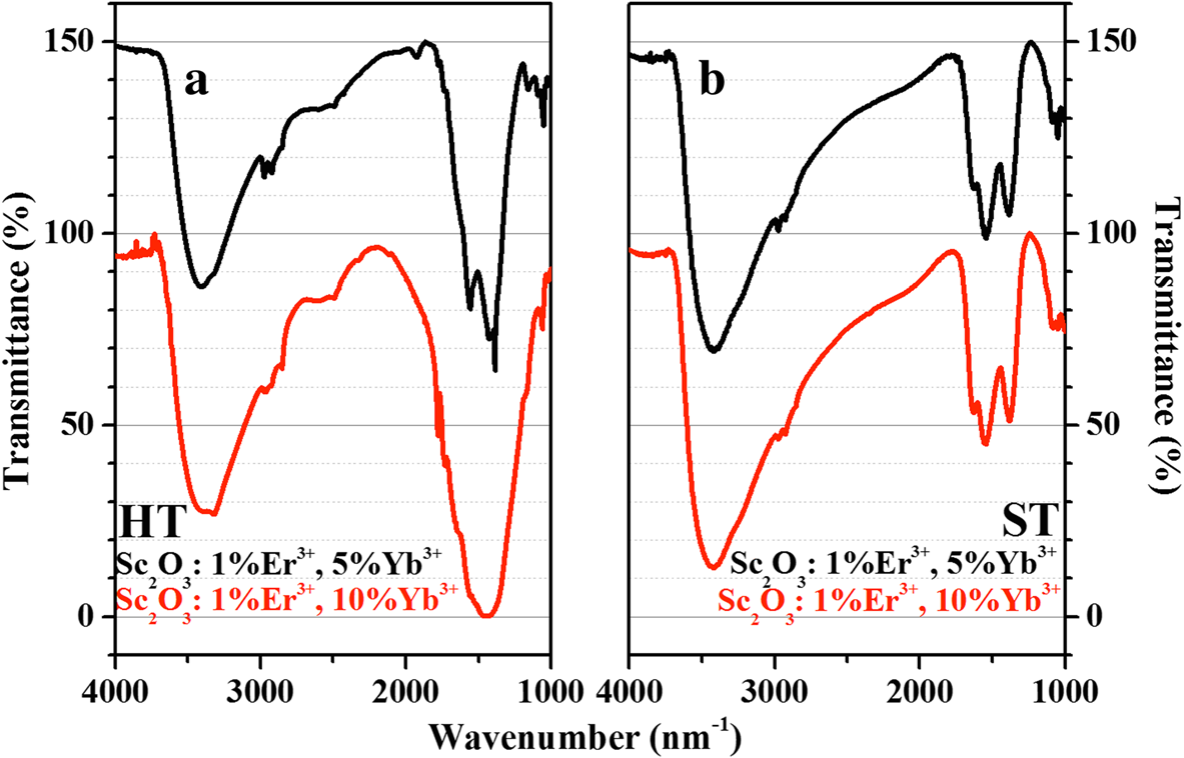
FTIR spectra of HT-Sc_2_O_3_: 1%Er^3+^, 5%/10%Yb^3+^ (**a**) and ST-Sc_2_O_3_: 1%Er^3+^, 5%Yb^3+^/10%Yb^3+^ (**b**) samples

The FTIR spectra of HT-Sc_2_O_3_: 1%Er^3+^, 5%/10%Yb^3+^ and ST-Sc_2_O_3_: 1%Er^3+^, 5%Yb^3+^/10%Yb^3+^ samples are shown in Fig. 4. The broad band around 3429 cm^− 1^ is attributed to the stretching vibration of –OH in the oleic acid (OA) and water [26, 27]. The 2925 and 2850 cm^− 1^ absorption bands are assigned to the asymmetric and symmetric stretching vibrations of the methylene (CH_2_) in the long alkyl chain of the OA molecules. The sharpness of the bands indicates that the hydrocarbon chains are well ordered. The anti-symmetric methyl stretch (CH_3_) is seen as a shoulder on the peak at 2975 cm^−1^. The bands at 1200–1750 cm^−1^ can be assigned to the vibrations of C=O in the oleic acid molecule and CO_2_ in the air [28]. The transformation to carbonate might have occurred on the surface of crystallites during the heat treatment. These results evidence the existence of capping ligands on the surfaces of samples. Figure 4 shows the absorption intensities of –OH vibration for ST-Sc_2_O_3_ samples are stronger. The intensities of surface groups for HT/ST-Sc_2_O_3_: 1%Er^3+^, 10%Yb^3+^ samples are both stronger than that in co-doped 5%Yb^3+^ samples. The abundant surface groups with available large vibrational quanta may efficiently enhance the MPR processes, inducing the decline of luminescence.

In order to exactly describe the population mechanism in Er^3+^/Yb^3+^ co-doped HT-Sc_2_O_3_ sample, the dependence of spectral distributions on the Er^3+^/Yb^3+^ concentrations has been studied in detail.

**Fig. 5:**
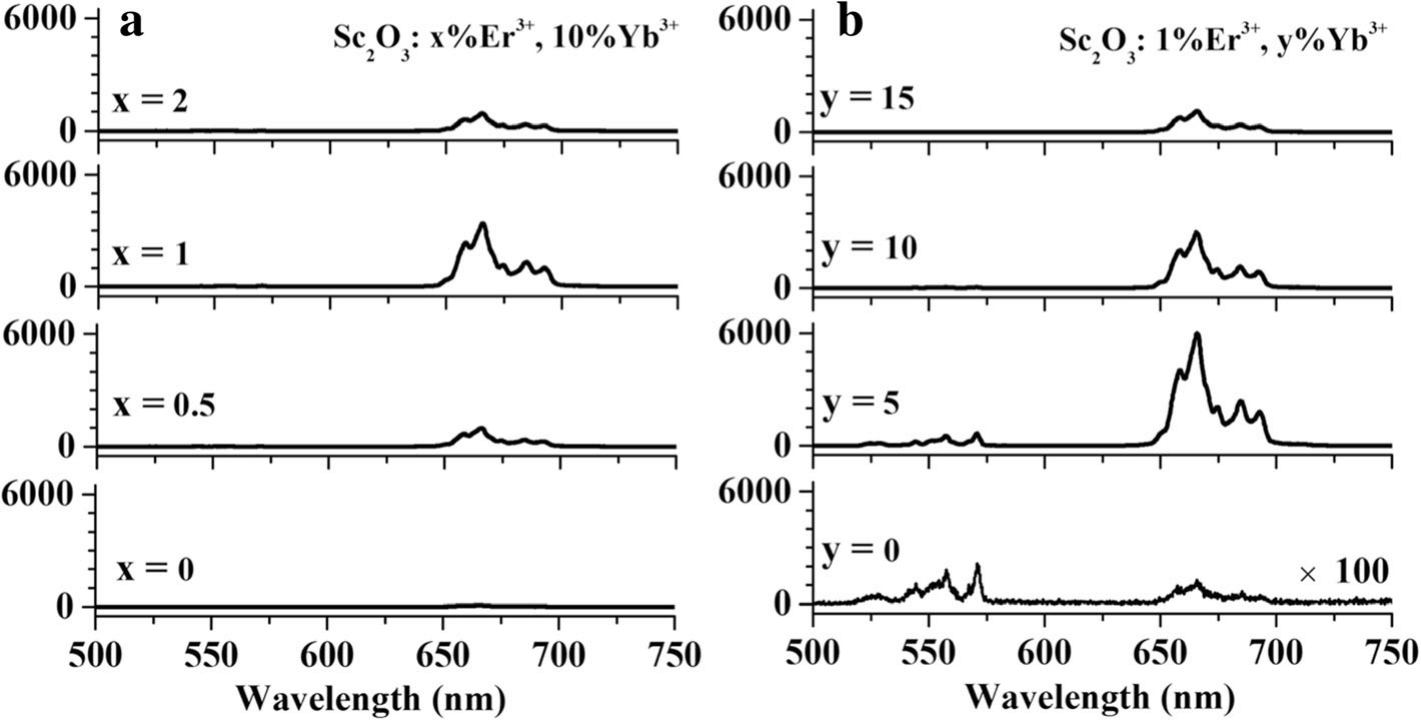
UCL spectra of HT-Sc_2_O_3_: x%Er^3+^, 10%Yb^3+^ (*x* = 0, 0.5, 1, 2) (**a**) and HT-Sc_2_O_3_: 1%Er^3+^, y%Yb^3+^, (*y* = 0, 5, 10, 15) (**b**) under 980 nm excitation

The UCL spectra of HT-Sc_2_O_3_: x%Er^3+^, 10%Yb^3+^ (*x* = 0, 0.5, 1, 2) under 980 nm excitation are presented in Fig. 5a. For the fixed Yb^3+^ concentration at 10%, the strongest UCL is observed for Er^3+^ concentration around 1%. When Er^3+^ concentration exceeds 1%, the intensity begins to diminish because of the cross relaxation (CR) of Er^3+^ ions [17]. The UCL spectra of HT-Sc_2_O_3_: 1%Er^3+^, y%Yb^3+^, (*y* = 0, 5, 10, 15) are presented in Fig. 5b. For the Er^3+^ singly doped Sc_2_O_3_, its UC emission is very faint, which has been magnified 100 times. The ET process of Yb^3+^ → Er^3+^ plays a dominant role for UCL enhancement. The strongest UCL is observed for Yb^3+^ concentration 5% when fixed the optimal Er^3+^ concentration 1%.

**Fig. 6:**
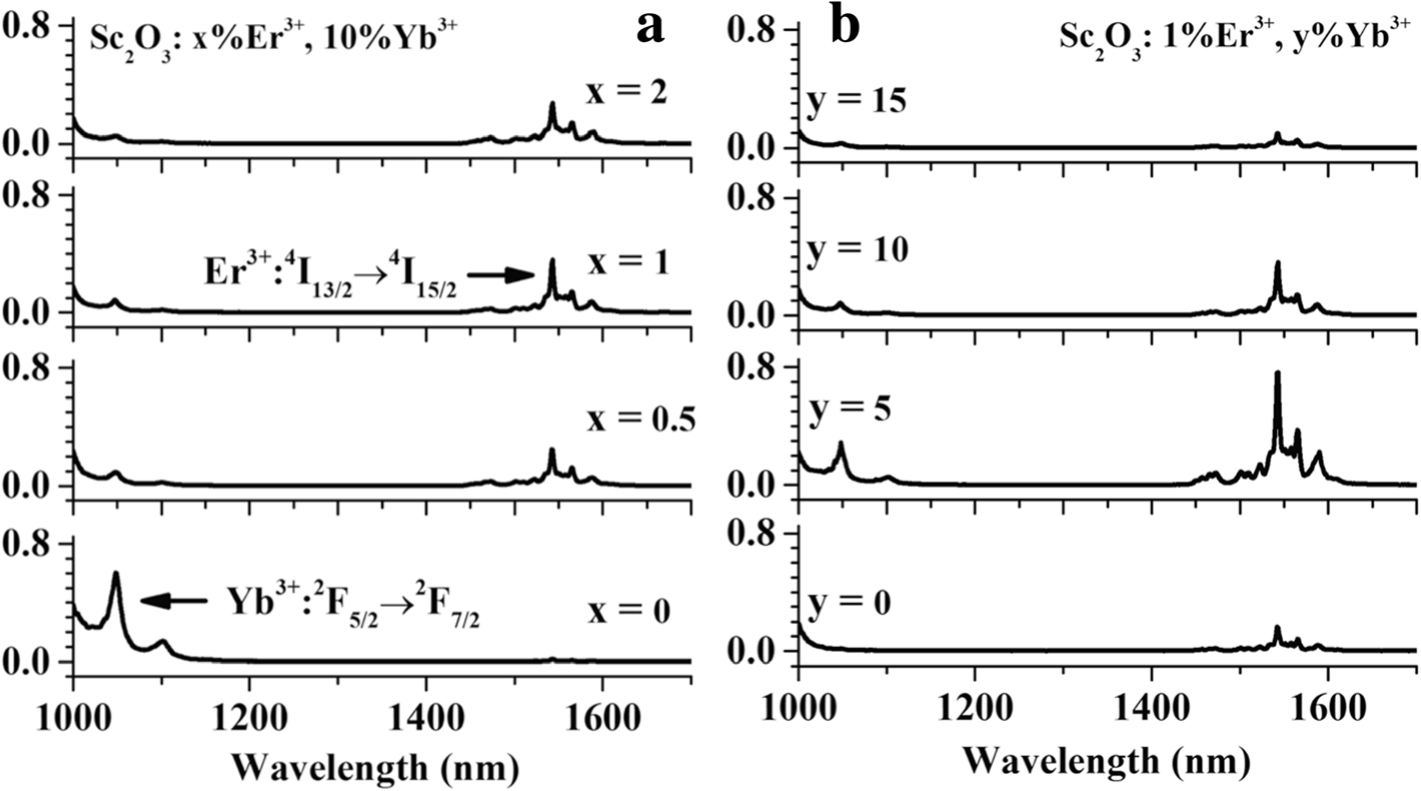
Near-infrared emission spectra in the range of 1000–1700 nm for HT-Sc_2_O_3_: x%Er^3+^, 10%Yb^3+^ (*x* = 0, 0.5, 1, 2) (**a**) and HT-Sc_2_O_3_: 1%Er^3+^, y%Yb^3+^, (*y* = 0, 5, 10, 15) (**b**) under 980 nm excitation

The near infrared emission spectra in the range of 1000–1700 nm for the same variety samples are shown in Fig. 6. In the Er^3+^/Yb^3+^ co-doped samples, 980-nm photon excites Yb^3+^: ^2^F_7/2_ → ^2^F_5/2_ which exhibits fluorescence at 1000–1200 nm exciting Er^3+^ ions into ^4^I_11/2_ level through a nonresonant phonon-assisted ET process [9]. The Er^3+^ ions in ^4^I_11/2_ level decay nonradiatively to ^4^I_13/2_ level, then radiatively to the ground state emitting the photon around 1550 nm [9]. In Fig. 6a, as Er^3+^concentration increases, the Yb^3+^ emission has a steady decline which evidences the efficient Yb^3+^ → Er^3+^ ET. The Er^3+^ emission gradually increases when Er^3+^ concentration increases from 0 to 1%, then declines slightly as a result of the self-absorption of Er^3+^ ions. In Fig. 6b, Er^3+^: ^4^I_13/2_ emission gradually enhances when Yb^3+^ concentration increases from 0 to 5% but subsequently begins to decrease. As Yb^3+^ concentration increases, Yb^3+^ capacity of 980 nm photon absorption is enhanced. The Yb^3+^ emission intensity is shown to increase. Meantime, as the distance of Yb-Yb and Yb-Er pairs decreases, the enhanced energy migration among Yb^3+^ ions speeds up ET from Yb^3+^ to Er^3+^. It leads to the increased population of Er^3+^: ^4^I_13/2_ level but the decreased one of Yb^3+^: ^2^F_5/2_level. Due to the quenching of Er^3+^ by Yb^3+^ ions, the emission of Er^3+^: ^4^I_13/2_ → ^4^I_15/2_ reaches a maximum then drops down.

**Fig. 7:**
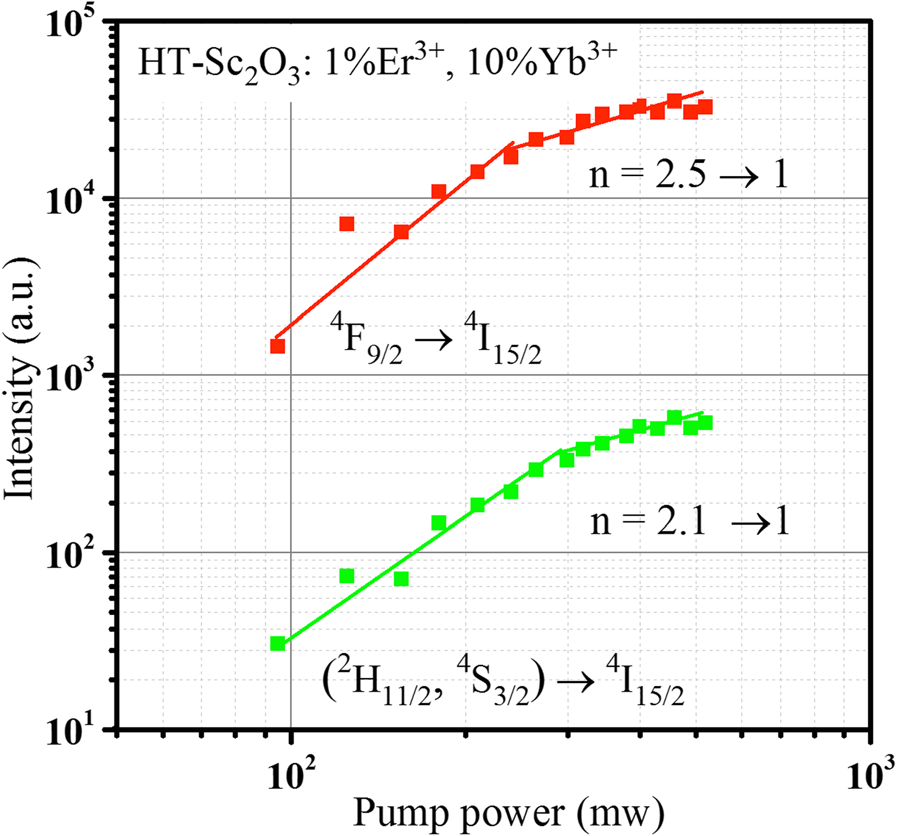
Power dependence curves for Er^3+^: (^2^H_11/2_, ^4^S_3/2_) → ^4^I_15/2_ and ^4^F_9/2_ → ^4^I_15/2_ transitions in HT-Sc_2_O_3_: 1%Er^3+^, 10%Yb^3+^ NPs

The pumping power dependences of Er^3+^: (^2^H_11/2_, ^4^S_3/2_) → ^4^I_15/2_ and Er^3+^: ^4^F_9/2_ → ^4^I_15/2_ intensities in HT-Sc_2_O_3_: 1%Er^3+^, 10%Yb^3+^ are measured under 980 nm excitation and plotted in a double logarithmic scales in Fig. 7. For the UCL processes, the UCL intensity (*I*_UCL_) depends on the pumping laser power (*P*) as the equation: *I*_*UCL*_ ∝ *Pn* where *n* is the number of pumping photons absorbed per upconverted photon emitted [29]. The *n* value can be obtained from the slope of the linear plots between log (*I*) and log (*P*). For the two-step ET process, the *n* value is theoretically less than 2 due to the competition between linear decay and UC processes. Figure 7 shows the slope *n* values for red and green emissions are 2.5 and 2.1 in the low pump power density, respectively. It indicates, except for two-step process, that there are also the three-photon processes in HT-Sc_2_O_3_: 1%Er^3+^, 10%Yb^3+^ NPs [30, 31].

**Fig. 8:**
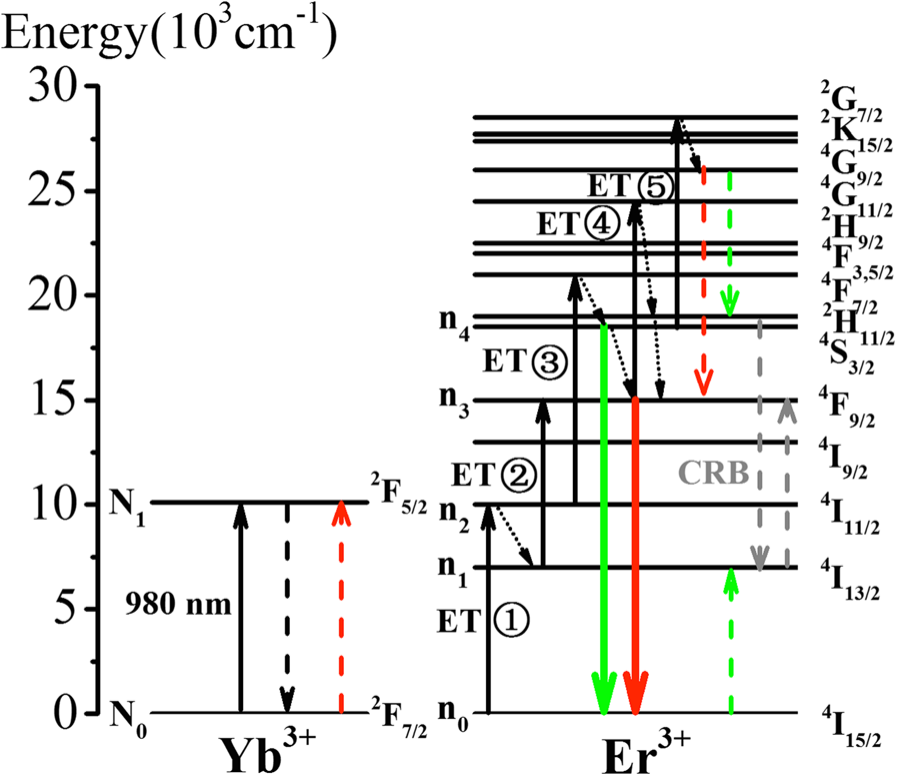
The energy level diagrams and dominant upconversion mechanism in Sc_2_O_3_: Er^3+^, Yb^3+^ NPs under 980 nm pump

The upconversion mechanism is drawn in Fig. 8. The ET process is as follows:

- The ET①: Yb^3+^: ^2^F_5/2_ + Er^3+^: ^4^I_15/2_ → Yb^3+^: ^2^F_7/2_ + Er^3+^: ^4^I_11/2_
  - Er^3+^: ^4^I_11/2_ → Er^3+^: ^4^I_13/2_ (MPR)
- The ET②: Yb^3+^: ^2^F_5/2_ + Er^3+^: ^4^I_13/2_ → Yb^3+^: ^2^F_7/2_ + Er^3+^: ^4^F_9/2_
- The ET③: Yb^3+^: ^2^F_5/2_ + Er^3+^: ^4^I_11/2_ → Yb^3+^: ^2^F_7/2_ + Er^3+^: ^4^F_7/2_
  - Er^3+^: ^4^F_7/2_ → Er^3+^: (^2^H_11/2_, ^4^S_3/2_) (MPR)
- The ET④: Yb^3+^: ^2^F_5/2_ + Er^3+^: ^4^F_9/2_ → Yb^3+^: ^2^F_7/2_ + Er^3+^: ^2^H_9/2_
  - Er^3+^: ^2^H_9/2_ → Er^3+^: (^2^H_11/2_, ^4^S_3/2_) (MPR)
  - Er^3+^: (^2^H_11/2_, ^4^S_3/2_) → Er^3+^: ^4^F_9/2_ (MPR)
- The ET⑤: Yb^3+^: ^2^F_5/2_ + Er^3+^: (^2^H_11/2_, ^4^S_3/2_) → Yb^3+^: ^2^F_7/2_ + Er^3+^: ^2^G_7/2_

To verify and make a theoretical interpretation of the UCL results mentioned above, we utilize the simplified steady-state equations.

1 $$ \frac{dn_0}{dt}=0 $$

2 $$ \frac{dn_1}{dt}={n}_2{W}_{21}-{C}_2{N}_1{n}_1-\frac{n_1}{\tau_1} $$

3 $$ \frac{dn_2}{dt}={C}_1{N}_1{n}_0-{C}_3{N}_1{n}_2-{n}_2{W}_{21}-\frac{n_2}{\tau_2} $$

4 $$ \frac{dn_3}{dt}={C}_2{N}_1{n}_1-{C}_4{N}_1{n}_3-\frac{n_3}{\tau_3} $$

5 $$ \frac{dn_4}{dt}={C}_3{N}_1{n}_2-{C}_5{N}_1{n}_4-\frac{n_4}{\tau_4} $$

6 $$ \frac{dN_1}{dt}=\sigma {IN}_0-{C}_1{N}_1{n}_0-{C}_2{N}_1{n}_1-{C}_3{N}_1{n}_2-{C}_4{N}_1{n}_3-{C}_5{N}_1{n}_4-\frac{N_1}{\tau_{Yb}}=0 $$

Where *σ* is the absorption cross section of Yb^3+^ ions, *I* is the incident pumping power, *N*_*i*_ is the population density of the *i*th level of Yb^3+^, *n*_*i*_ is the population density of *i*th level of Er^3+^ involved in the upconversion process, *τ*_i_ is the lifetime of *i*th level of Er^3+^and *τ*_Yb_ is the lifetime of ^2^F_5/2_ level of Yb^3+^, *C*_*i*_ represents the ET coefficient of Yb^3+^ → Er^3+^ for steps *i* = 1, 2, 3, 4, 5, and *W*_21_ represents the nonradiative rate between 1 and 2 levels of the Er^3+^ ions.

Compared with two-step process, the UC efficiency of three-photon processes from NIR to visible is decreased [32]. Additionally, the high-photon process is prominent when pumping power is high enough. The excitations of Er^3+^: ^4^F_9/2_ by ET to Er^3+^: ^2^H_9/2_ can be neglected due to the weak pump in our experiment. By Eq. (4), the red emission intensity (*I*_Red_) can be obtained by

$$ {I}_{Red}={\gamma}_3{n}_3={\gamma}_3{C}_2{\tau}_3{I}_{Yb}{I}_{n_1} $$

**Fig. 9:**
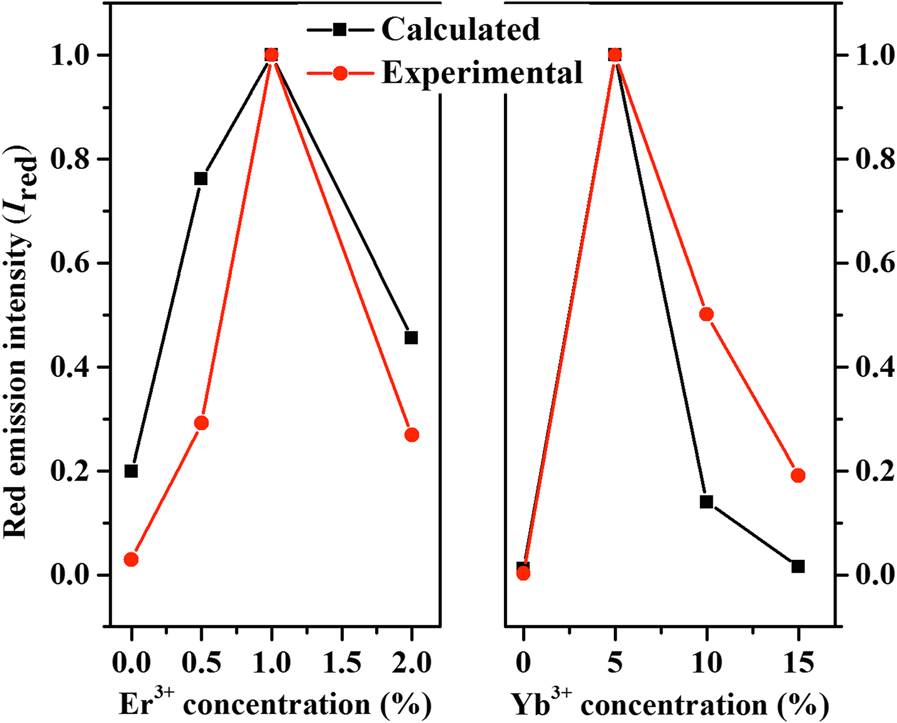
Calculated and experimental red emission intensities (*I*_Red_) values at various Er^3+^/Yb^3+^concentrations. The intensities are scaled to the maximum

Due to the CR of the Er^3+^– Er^3+^ interaction is not considered, the lifetime, *τ*_3_, is a constant. That is to say, ${\mathrm{I}}_{\mathrm{Red}}\propto {\mathrm{I}}_{\mathrm{Yb}}{\mathrm{I}}_{{\mathrm{n}}_1}$, where *I*_Yb_ and *I*_n1_ represent the emission intensity of Yb^3+^: ^2^F_5/2_ and Er^3+^: ^4^I_13/2_, respectively. The γ_3_ is radiative rate of red emission. The calculated *I*_Red_values at various Er^3+^/Yb^3+^concentrations are presented in Fig. 9, scaled to the maximum. For comparison, the *I*_Red_ values obtained directly from the UCL emission spectra are also depicted. The calculated and experimental *I*_Red_ trends are consistent with each other and obtain the best value at the same Er^3+^/Yb^3+^ concentrations, demonstrating the validity of experimental data.

The three-photon green and red UC processes occurred simultaneously result in the increase of the corresponding *n* values. Meanwhile, the *n* value of red UC process increases more effectively than that of green UC process. In Fig. 8, the green and red UCL can be populated by CR, as Er^3+^: ^4^G_11/2_ + Er^3+^: ^4^I_15/2_ → Er^3+^: (^2^H_11/2_, ^4^S_3/2_) + Er^3+^: ^4^I_13/2_ and Er^3+^: ^4^G_11/2_ + Yb^3+^: ^2^F_7/2_ → Er^3+^: ^4^F_9/2_ + Yb^3+^: ^2^F_5/2_, respectively [31]. The three-photon green UCL is via a cross-relaxation process between two Er^3+^ ions; however, the cross-relaxation in the three-photon red UCL is between Yb^3+^ and Er^3+^ ions. Since the Yb^3+^ concentration is much higher than Er^3+^ in our experiment, the three-photon red UC process is more effective than the three-photon green UC process, resulting in a rapid increase of *n* value for red UCL. In addition, it should be noted that all the three-photon processes are few, so the *n* values deviate obviously from 3. At the high pump power density, two slopes gradually drop to 1 because UC process becomes dominant [33].

**Fig. 10:**
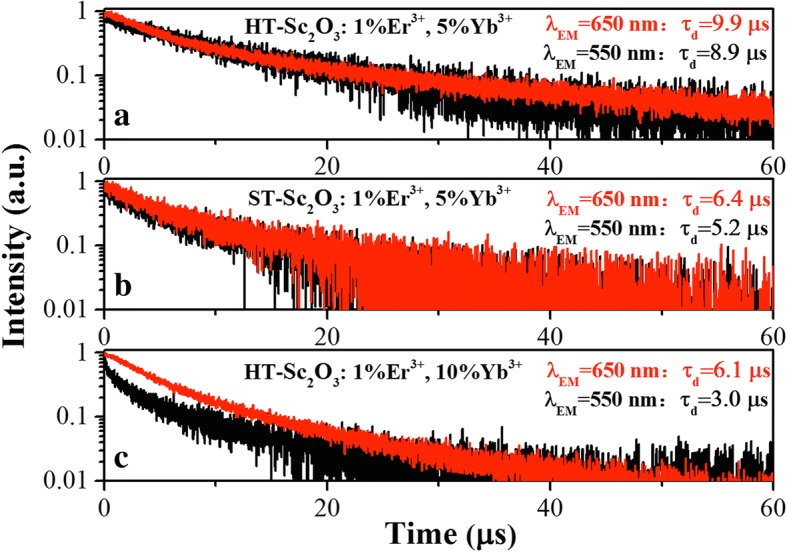
Decay curves of the Er^3+^: (^2^H_11/2_, ^4^S_3/2_) → ^4^I_15/2_ and ^4^F_9/2_ → ^4^I_15/2_ transitions in HT-Sc_2_O_3_ and ST-Sc_2_O_3_ samples under the 980 nm excitation wavelength

The decay curves of the Er^3+^: (^2^H_11/2_, ^4^S_3/2_) → ^4^I_15/2_ and ^4^F_9/2_ → ^4^I_15/2_ transitions in HT-Sc_2_O_3_ and ST-Sc_2_O_3_ samples under the 980 nm excitation wavelength have been measured and shown in Fig. 10. The decay times for red and green emissions are calculated by integrating the area under the corresponding decay curves with the normalized initial intensity. Figure 10a, b shows the green and red emission lifetimes in HT-Sc_2_O_3_: 1%Er^3+^, 5%Yb^3+^ are longer than those in ST-Sc_2_O_3_: 1%Er^3+^, 5%Yb^3+^. The lifetime is proportional to population of level. The longer values indicate the stronger red and green UCL in HT-Sc_2_O_3_ sample. In our previous report, we found our samples own the shorter decay lifetime values than that in the literature. Actually, the decay times of Er^3+^: (^2^H_11/2_, ^4^S_3/2_) → ^4^I_15/2_ and ^4^F_9/2_ → ^4^I_15/2_ emissions for HT/ST-Sc_2_O_3_: 1%Er^3+^, 5%Yb^3+^ samples are both close to each other. If Er^3+^: ^4^F_9/2_ level is populated by the MPR process from Er^3+^: (^2^H_11/2_, ^4^S_3/2_) levels, the decay time of Er^3+^: ^4^F_9/2_ level approaches to that of Er^3+^: ^4^S_3/2_ level. However, this MPR process is inefficient for population of Er^3+^: ^4^F_9/2_ level [17]. There is another non-MPR mechanism for populating the Er^3+^: ^4^F_9/2_level from Er^3+^: ^4^S_3/2_ level. The mechanism involves CR ET: Er^3+^: (^2^H_11/2_, ^4^S_3/2_) + Yb^3+^: ^2^F_7/2_ → Er^3+^: ^4^I_13/2_ + Yb^3+^: ^2^F_5/2_; then, in the same Er^3+^–Yb^3+^ pair, an energy back transfer (CRB) Yb^3+^: ^2^F_5/2_ + Er^3+^: ^4^I_13/2_ → Yb^3+^: ^2^F_5/2_ + Er^3+^: ^4^F_9/2_occurs [1]. If the CRB process dominates the main way for the population of Er^3+^: ^4^F_9/2_ level, the decay time of Er^3+^: ^4^F_9/2_ level should be almost equal to the decay time of Er^3+^: ^4^S_3/2_ level. The CRB process is fast and efficient at low excitation density.

**Fig. 11:**
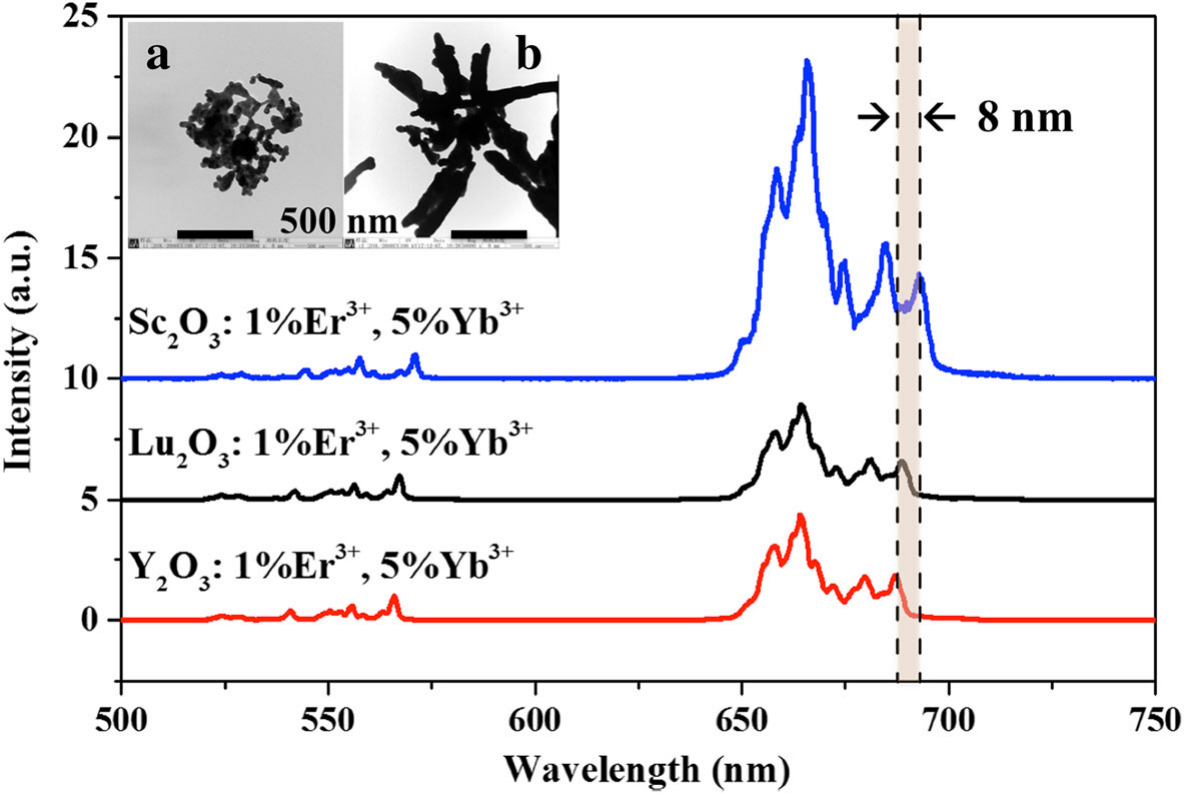
UCL spectra of Er^3+^/Yb^3+^ co-doped typical sesquioxide materials under 980 nm excitation

Figure 11 shows the UCL spectra of three typical sesquioxides under 980 nm excitation. The Sc_2_O_3_: 1%Er^3+^, 5%Yb^3+^ sample exhibits the strongest UCL in the series of spectra. Furthermore, the emission line of Er^3+^: ^4^F_9/2_ level at the lowest energy side in Sc_2_O_3_ shifts to the longer wavelength side by 8 nm relative to that in Y_2_O_3_. The nearest Sc-Sc distance is 3.27 Å in Sc_2_O_3_ shorter than the Y-Y distance (3.752 Å) in Y_2_O_3_ [3, 17]. The mean Sc–O bond length (2.121 Å) in Sc_2_O_3_ is shorter than the mean Y–O bond length (2.263 Å) in Y_2_O_3_. The Er^3+^/Yb^3+^ on Sc^3+^ site in Sc_2_O_3_ experiences a stronger crystal field than on Y^3+^ site in Y_2_O_3_. The red shift of spectrum can be attributed to the large Stark splitting of Er^3+^ ions in Sc_2_O_3_ host. The morphologies of Y_2_O_3_ and Lu_2_O_3_ samples were also characterized by TEM as shown in the inset of Fig. 11a, b, respectively, for comparison. The obtained spherical particles are both agglomerated to bulk. The better dispersion and uniformity of Sc_2_O_3_ NPs synthesized by HT method favor its application in biological assays and medical image.

## Conclusions

In summary, Sc_2_O_3_: Er^3+^, Yb^3+^ NPs about 19 nm were synthesized by a simple oleic acid-mediated HT process. The Sc_2_O_3_: Er^3+^, Yb^3+^ NPs by HT method shows the stronger UCL, of which the red UCL are enhanced by a factor of 4, in comparison with that in the same optimized concentration Sc_2_O_3_ samples by ST method. The UCL enhancement can be attributed to the reduced surface groups and longer lifetimes. The surface groups enhanced the MPR, inducing the decline of luminescence. Under the 980 nm excitation, the decay curves of Er^3+^: (^2^H_11/2_, ^4^S_3/2_) → ^4^I_15/2_ and ^4^F_9/2_ → ^4^I_15/2_ emissions for HT-Sc_2_O_3_: 1%Er^3+^, 5%Yb^3+^ samples are close to each other, resulting from the non-MPR mechanism for populating the Er^3+^: ^4^F_9/2_ level from Er^3+^: ^4^S_3/2_ level. The mechanism involves CR ET: Er^3+^: (^2^H_11/2_, ^4^S_3/2_) + Yb^3+^: ^2^F_7/2_ → Er^3+^: ^4^I_13/2_ + Yb^3+^: ^2^F_5/2_; then, in the same Er^3+^–Yb^3+^ pair, an energy back transfer (CRB) Yb^3+^: ^2^F_5/2_ + Er^3+^: ^4^I_13/2_ → Yb^3+^: ^2^F_5/2_ + Er^3+^: ^4^F_9/2_ occurs. Under the relatively low-power density, the slopes of the linear plots of log(*I*) vs log(*P*) for red and green emissions are 2.5 and 2.1, respectively, which are larger than 2 because of the existence of three-photon processes. Compared with the typical sesquioxides (Y_2_O_3_ and Lu_2_O_3_), the Sc_2_O_3_: 1%Er^3+^, 5%Yb^3+^ NPs exhibits the stronger UCL. Furthermore, in Sc_2_O_3_ the emission line of Er^3+^: ^4^F_9/2_ level at the lowest energy side shifts to the longer wavelength side by 8 nm relative to that in Y_2_O_3_ owing to the large Stark splitting of Er^3+^ ions in Sc_2_O_3_ host. Results show the Sc_2_O_3_: Er^3+^, Yb^3+^ nanoparticles (NPs) is an excellent material for achieving intense UCL with small size in the biological fields.

## Abbreviations

CR: Cross relaxation
ET: Energy transfer
HT: Hydrothermal
NCs: Nanocrystals
NPs: Nanoparticles
OPO: Optical parametric oscillator
ST: Solvothermal
TEM: Transmission electron microscopy
UCL: Upconversion luminescence
XRD: X-ray diffraction
